# Analyzing Th17 cell differentiation dynamics using a novel integrative modeling framework for time-course RNA sequencing data

**DOI:** 10.1186/s12918-015-0223-6

**Published:** 2015-11-17

**Authors:** Jukka Intosalmi, Helena Ahlfors, Sini Rautio, Henrik Mannerstöm, Zhi Jane Chen, Riitta Lahesmaa, Brigitta Stockinger, Harri Lähdesmäki

**Affiliations:** Department of Computer Science, Aalto University, Aalto, FI-00076 Finland; The Francis Crick Institute, Mill Hill Laboratory, Mill HillLondon, UK; Turku Centre for Biotechnology, University of Turku and Åbo Akademi, Turku, Finland; Current affiliation: Lymphocyte Signalling and Development, The Babraham Institute, Cambridge, UK

**Keywords:** T cell differentiation, T helper 17 cell, Sequencing data, Mathematical modeling, Statistical modeling, Computational statistics

## Abstract

**Background:**

The differentiation of naive CD 4^+^ helper T (Th) cells into effector Th17 cells is steered by extracellular cytokines that activate and control the lineage specific transcriptional program. While the inducing cytokine signals and core transcription factors driving the differentiation towards Th17 lineage are well known, detailed mechanistic interactions between the key components are poorly understood.

**Results:**

We develop an integrative modeling framework which combines RNA sequencing data with mathematical modeling and enables us to construct a mechanistic model for the core Th17 regulatory network in a data-driven manner.

**Conclusions:**

Our results show significant evidence, for instance, for inhibitory mechanisms between the transcription factors and reveal a previously unknown dependency between the dosage of the inducing cytokine TGF *β* and the expression of the master regulator of competing (induced) regulatory T cell lineage. Further, our experimental validation approves this dependency in Th17 polarizing conditions.

**Electronic supplementary material:**

The online version of this article (doi:10.1186/s12918-015-0223-6) contains supplementary material, which is available to authorized users.

## Background

Adaptive immunity is largely mediated by CD 4^+^ T cells which are a subclass of lymphocytes [1]. When a naive CD 4^+^ T cell encounters an antigen in the presence of cytokine signals, the cell is activated through T cell receptor and the differentiation into one of the effector T helper (Th) cell subsets can be initiated [1]. There are four well characterized subsets of CD 4^+^ T cells, Th1, Th2, Th17, and induced regulatory T (iTreg) cells, all of them having distinct functions in the adaptive immune system [1]. The subset of Th17 cells is the most recently discovered subset and, consequently, there has been a keen interest in studying the properties of this subset during the past decade [2–5]. The primary function of Th17 cells is to clear pathogens during host defense reactions and, in general, Th17 cells have been noted to have important functions in various autoimmune diseases such as psoriasis, rheumatoid arthritis, multiple sclerosis, asthma, and inflammatory bowel disease [4, 5].

The differentiation from a naive CD 4^+^ T cell into a Th17 cell is induced by two cytokines, transforming growth factor *β* (TGF *β*) and interleukin 6 (IL6) and both of these cytokines are required for a successful differentiation [4, 6]. The key transcription factors for Th17 cell generation are the retinoic acid receptor-related orphan receptor gamma t (ROR *γ*t) and signal transducer and activator of transcription 3 (STAT3) [4, 6]. In the course of the differentiation, STAT3 is activated through an extracellular IL6 signal and, in the presence of a sufficient TGF *β* level, STAT3 induces ROR *γ*t expression which leads to Th17 differentiation and interleukin 17 secretion [4, 6]. Th17 cell differentiation is also closely related to the generation of iTreg cells. The relation between iTreg and Th17 lineages is reciprocal in nature, differentiation into both subtypes requires a sufficient amount of TGF *β*, and these subtypes have also been shown to exhibit plasticity [4, 7–9]. Recent experimental studies provide a plethora of information about the Th17 lineage specific regulatory network [6, 10] but precise mechanistic understanding of the transcription factor dynamics is yet to be attained. In this study, we take the first steps towards a mechanistic characterization of the Th17 specific core regulatory network by using mathematical modeling that is employed to extract information from time-course measurements of mRNA kinetics.

Mathematical modeling can be used to construct detailed descriptions for kinetics of molecular processes and, consequently, it offers rigorous and objective means to test whether the current understanding about the molecular system is in agreement with observed data or otherwise expected behavior. During the past years, mathematical models have been developed to study the differentiation from naive CD 4^+^ T cells into Th1, Th2, Th17, and iTreg subsets [11–22]. Some of the published models describe the differentiation into several distinct subsets and, in the context of lineage specification, these models describe also the differentiation into Th17 subset [17, 18, 20–22]. However, to our best knowledge, a detailed dynamic model describing the dynamics of core transcription factors behind Th17 cell differentiation has not been published. Developing this kind of detailed model is timely because recent experimental studies provide us with sufficient amount of information about the dependencies between the key transcription factors and, on the other hand, learning the mechanistic underpinnings of regulatory relationships during the differentiation process is crucial, for instance, for the design of experimental approaches to modulate the immune response.

In this study, we construct a dynamic description for the core molecular mechanisms steering Th17 cell differentiation and use mathematical modeling to quantitatively predict the resulting molecular dynamics. The dynamic description is calibrated in a data-driven manner, and both the calibrated dynamic model and biological findings are further validated experimentally. Our analysis rests upon comprehensive time-course RNA sequencing measurements that provide us with a system level understanding of dynamic gene expression kinetics during the Th17 cell differentiation of primary murine T cells in vitro. In order to combine mathematical modeling with these data and enable experimentally-based modeling, we develop a statistical framework that is designed specifically for time-course RNA sequencing data and, further, we elaborate the statistical techniques that are essential for obtaining our results. Our treatment of sequencing data is based on the negative binomial distribution that is also used in the state-of-the-art statistical data analysis methods and this approach enables us to integrate mathematical modeling with sequencing data in a natural way.

In summary, we present a computational framework that is used to derive the first detailed dynamic model for the core network behind Th17 cell differentiation and, further, we validate the model experimentally. Our results show significant evidence, for instance, for inhibitory mechanisms between the transcription factors and also reveal a previously unknown dependency between the dosage of TGF *β* and the expression of the master regulator of competing iTreg lineage in Th17 polarizing conditions.

## Methods

### RNA-seq data

The flow cytometry sorted naive (CD 4^+^ CD44 ^low^ CD25 ^-^) T cells isolated from lymph nodes and spleens of C57BL/6 mice were activated with plate-bound anti-CD3 (0.5 ug/ml; 2C11 eBioScience) and anti-CD28 (5 ug/ml; 37.51; eBioScience) and cultured in Th17 conditions with IL6 (20 ng/ml) and TGF *β* (1 ng/ml) both mouse origin, R&D Systems. Cultures were performed in triplicates. The culture media used was IMDM (Sigma-Aldrich) supplemented with 5 % FCS, 2×10^−3^ M L-glutamine, 100 U/ml penicillin, 100 mg/ml streptomycin and 5×10^−5^ M b-mercaptoethanol (all Sigma). Samples were harvested at indicated time points. The sequencing data were preprocessed and analyzed as described below. The data used in this study (read counts, library sizes, and estimated dispersion parameters) are given in Additional file 1.

### FOXP3 protein data

Naive T cells were isolated and activated as described above. Cells were cultured in Th17 conditions with a constant concentration of IL6 (20 ng/ml) and five different concentrations of TGF *β* (1/16,1/8,1/4,1/2, and 1 ng/ml) both mouse origin, R&D Systems. Cultures were performed in triplicates using culture media described above. At day 3, the cells were stimulated with PdBu and ionomycin and stained for intracellular FOXP3.

### Data analysis

Sequence reads were mapped using Tophat (version 1.3.2) with default parameters to the NCBIM37 mouse reference genome and Ensembl mouse transcriptome (release 63). Expression levels were estimated for all the Ensembl genes using Python script rpkmforgenes with parameters -readcount -no3utr [23], which ignores 3’ UTRs. Bioconductor package edgeR [24] was used to estimate the dispersion parameters. Dispersion estimation was done for each time point separately and by taking into account the paired experimental design.

### The core network

**Fig. 1:**
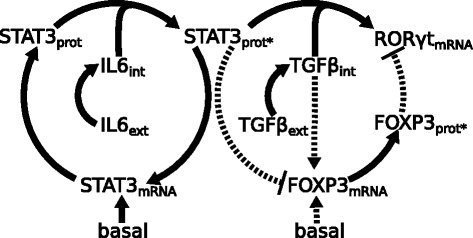
Schematic illustration of the dynamic description. Illustration shows the assumed (solid connectors) and hypothetical (dashed connectors) interactions during Th17 lineage specification

The differentiation of naive CD 4^+^ T cells into effector Th17 cells is a highly complex process which is affected by a large number of interacting molecules and many of the regulated interactions are still unknown [4, 6, 10]. However, it is intriguing that the key transcription factors such as the master regulator ROR *γ*t and STAT3 show clear dynamics during Th17 polarization. Given these strong lineage specific dynamics, we hypothesize that a simplified view on the differentiation kinetics can be captured by observing the time evolution of the core components driving the differentiation. In order to implement these ideas, we construct a dynamic description for the core Th17 network based on the current literature and test the description quantitatively against experimental data by means of statistical analysis. Here, the use of mathematical modeling is crucial because a direct statistical analysis of the time-course data is not capable of capturing the dependencies and dynamics that originate from the molecular kinetics. Our dynamic description for the core network involves the inducing cytokine signals, IL6 and TGF *β* and the transcription factors ROR *γ*t, STAT3, and FOXP3 (see the schematic illustration in Fig. 1). The mechanistic reasoning for the core network is based on the central dogma, mRNA molecules are translated into proteins which in turn are phosphorylated and regulate the expression of their target genes. The master regulator of induced regulatory T (iTreg) cells, FOXP3 is included to allow possible balancing effects of this competing lineage.

Th17 lineage specification is highly dependent on the inducing extracellular cytokine signals IL6 and TGF *β*. In our dynamic description, we treat the amount of added cytokines, that is the initial levels of extracellular cytokines, as the input signals. When activated CD 4^+^ T cells are exposed to these signals, the intracellular cytokine levels gradually start to increase and the Th17 lineage specific differentiation program becomes activated. The IL6 signal has a central role as it participates in the phosphorylation of STAT3 protein [4] and phosphorylated STAT3 protein activates the master regulator ROR *γ*t together with TGF *β* (Fig. 1). Along with activating ROR *γ*t, the phosphorylated STAT3 protein affects STAT3 expression in an autoregulatory manner. This feedback describes implicitly all autoregulatory mechanisms affecting STAT3 as well as possible autocrine STAT3 activation that might occur via different cytokine signals, for instance, interleukin 21 [4]. Experimental studies show that ROR *γ*t does not participate in the regulatory feedback mechanisms of STAT3 [6] and, consequently, we do not allow ROR *γ*t to affect STAT3 dynamics.

In addition to the mechanisms described above, we also include four additional regulatory mechanisms in our dynamic description. These mechanisms are hypothetical in the sense that it is not clear if they play significant roles in controlling the transcriptional dynamics in Th17 polarizing conditions. The hypothetical mechanisms include (i) basal activation of FOXP3, (ii) FOXP3 activation by TGF *β* [1], (iii) ROR *γ*t inhibition by FOXP3 [4], and (iv) FOXP3 inhibition by STAT3 [1]. The schematic illustration in Fig. 1 highlights these interactions using dashed connectors. By including different subsets of the hypothetical mechanisms into the core network, we construct altogether 12 alternative scenarios for regulatory mechanisms (Table 1). In order to analyze the observed mRNA dynamics by objective means, we convert our schematic description into a rigorous mathematical model and use experimental data and statistical testing to quantitatively assess the amount of evidence for each alternative scenario.

**Table 1:** Construction of alternative models

	M _1_	M _2_	M _3_	M _4_	M _5_	M _6_	M _7_	M _8_	M _9_	M _10_	M _11_	M _12_
Basal FOXP3 induction	×	×	×	×	–	–	–	–	×	×	×	×
TGF *β* induces FOXP3	–	–	–	–	×	×	×	×	×	×	×	×
FOXP3 inhibits ROR *γ*t	–	–	×	×	–	–	×	×	–	–	×	×
STAT3 inhibits FOXP3	–	×	–	×	–	×	–	×	–	×	–	×

Alternative models (M _*i*_, *i*=1,…,12) are obtained by considering different combinations of hypothetical interactions. Here, active and inactive interactions are denoted by × and –, respectively

### Mathematical model

We use ordinary differential equations (ODEs) to construct a mechanistic model for the core network driving the Th17 cell differentiation process. The mathematical model is based on the description of the core network introduced above.

The cytokine IL6 is indispensable for successful Th17 cell differentiation. In our model, we discriminate the extracellular IL6 (IL6 _ext_) and intracellular IL6 (IL6 _int_) levels. At time *t*=0 h, when the activated cells are treated with cytokines, IL6 _ext_ has its maximum value and, as time evolves, it starts gradually turn into intracellular IL6. This conversion is modeled using the equations

(1) $$\begin{array}{*{20}l} \frac{d [\!\text{IL6}_{\text{ext}}] }{d t} &= -\theta_{1} [\!\text{IL6}_{\text{ext}}] \end{array} $$

(2) $$\begin{array}{*{20}l} \frac{d [\!\text{IL6}_{\text{int}}] }{d t} &= \theta_{1} [\!\text{IL6}_{\text{ext}}], \end{array} $$

where *θ*_1_ is an unknown conversion rate. Initial level of intracellular IL6 equals zero and the initial value of [ IL6_ext_] corresponds to the amount of added IL6 [ IL6_added_] scaled by the Th17 specific level of IL6 input [ IL6_input_] (in this study [ IL6_input_] takes the value of 20 ng/ml). This yields an analytical solution for intracellular IL6 dynamics

(3) $$ [\!\text{IL6}_{\text{int}}](t) = \frac{[\!\text{IL6}_{\text{added}}]}{[\!\text{IL6}_{\text{input}}]}\left(1 - e^{-\theta_{1}t}\right).  $$

In the context of Th17 cell differentiation, IL6 plays an important role as it participates in the activation of STAT3 which, in turn, together with TGF *β* activates the Th17 lineage specific master regulator ROR *γ*t [4]. In order to incorporate detailed STAT3 dynamics into our model, we construct dynamic descriptions for STAT3 transcription, translation and IL6 driven activation. In addition, we allow STAT3 autoregulation that implicitly describes several possible feedback mechanisms (e.g. amplification through IL21 signals and positive feedback loops through other genes [4, 6]). We denote the relative abundances of STAT3 mRNA, STAT3 protein, and STAT3 phosphoprotein by [ STAT3_mRNA_], [ STAT3_prot_], and $[\!\text {STAT3}^{*}_{\text {prot}}]$, respectively, and write the dynamic system describing their interactions in the following form

(4) $$\begin{array}{*{20}l} \frac{d [\!\text{STAT3}_{\text{mRNA}}] }{d t} &=\quad \theta_{2} \end{array} $$

(5) $$\begin{array}{*{20}l} &\quad +\theta_{3}[\!\text{STAT3}_{\text{prot}^{*}}] \end{array} $$

(6) $$\begin{array}{*{20}l} &\quad -\theta_{4}[\!\text{STAT3}_{\text{mRNA}}] \end{array} $$

(7) $$\begin{array}{*{20}l} \frac{d [\!\text{STAT3}_{\text{prot}}] }{d t} &=\quad \theta_{5}[\!\text{STAT3}_{\text{mRNA}}] \end{array} $$

(8) $$\begin{array}{*{20}l} & \quad -\theta_{6}[\!\text{IL6}_{\text{int}}] [\!\text{STAT3}_{\text{prot}}] \end{array} $$

(9) $$\begin{array}{*{20}l} &\quad -\theta_{7} [\!\text{STAT3}_{\text{prot}}] \end{array} $$

(10) $$\begin{array}{*{20}l} \frac{d [\!\text{STAT3}_{\text{prot}^{*}}] }{d t} &=\quad \theta_{6}[\!\text{IL6}_{\text{int}}] [\!\text{STAT3}_{\text{prot}}] \end{array} $$

(11) $$\begin{array}{*{20}l} &\quad -\theta_{8} [\!\text{STAT3}_{\text{prot}^{*}}], \end{array} $$

where *θ*_2_−*θ*_8_ are unknown rate parameters (see Table 2). Given the strong basal expression level of STAT3, we assume that the initial level of STAT3 protein is non-zero. Without losing any generality, we treat [ STAT3_prot_] as a unitless quantity and set its initial level to equal one. The initial level of STAT3 phosphoprotein is taken to be zero.

**Table 2:** Descriptions for the model parameters

Parameter	Description
*θ*_1_	conversion rate for IL6
*θ*_2_	basal expression, STAT3
*θ*_3_	autoregulation, STAT3
*θ*_4_	mRNA degradation, STAT3
*θ*_5_	translation, STAT3
*θ*_6_	phosphorylation, STAT3
*θ*_7_	protein degradation, STAT3
*θ*_8_	phosphoprotein degradation, STAT3
*θ*_9_	conversion rate for TGF *β*
*θ*_10_	ROR *γ*t activation by TGF *β* and STAT3
*θ*_11_	ROR *γ*t inhibition by FOXP3
*θ*_12_	ROR *γ*t degradation
*θ*_13_	basal expression, FOXP3
*θ*_14_	FOXP3 activation by TGF *β*
*θ*_15_	FOXP3 inhibition by STAT3
*θ*_16_	mRNA degradation, FOXP3
*θ*_17_	translation/phosphorylation, FOXP3
*θ*_18_	protein degradation, FOXP3

In addition to IL6, TGF *β* cytokine is also required for successful Th17 cell differentiation. We model TGF *β* dynamics in the same manner as IL6 dynamics i.e. we discriminate the extracellular TGF *β* (TGF *β*_ext_) and intracellular TGF *β* (TGF *β*_int_) and describe the conversion of extracellular TGF *β* to intracellular by means of the ODEs

(12) $$\begin{array}{*{20}l} \frac{d [\!\text{TGF\(\beta\)}_{\text{ext}}] }{d t} &= -\theta_{9} [\!\text{TGF\(\beta\)}_{\text{ext}}] \end{array} $$

(13) $$\begin{array}{*{20}l} \frac{d [\!\text{TGF\(\beta\)}_{\text{int}}] }{d t} &= \theta_{9} [\!\text{TGF\(\beta\)}_{\text{ext}}] \end{array} $$

where *θ*_9_ is an unknown conversion rate parameter. Here, the initial level of intracellular TGF *β* is taken to be zero and the initial value of [ TGF *β*_ext_] corresponds to the amount of added TGF *β*[ TGF *β*_added_] scaled by the Th17 specific level of TGF *β* input [ TGF *β*_input_] (in this study [ TGF *β*_input_] takes the value of 1 ng/ml). The analytical solution for intracellular TGF *β* level is

(14) $$ [\!\text{TGF\(\beta\)}_{\text{int}}](t) = \frac{[\!\text{TGF}\beta_{\text{added}}]}{[\!\text{TGF}\beta_{\text{input}}]}\left(1 - e^{-\theta_{9}t}\right).  $$

The master regulator of Th17 lineage is ROR *γ*t and thus it is one of the most interesting components in our model. Because the basal expression of ROR *γ*t can be assumed to be very low (initial mRNA level is low, see data in Additional file 1, Section 2), we do not include it into the model. Instead, the activation of ROR *γ*t is taken to be induced by TGF *β* and STAT3 phosphoprotein. We also allow FOXP3, the master regulator of the induced regulatory T cells, to inhibit ROR *γ*t. These molecular interactions are modeled using the following ODE

(15) $$\begin{array}{*{20}l} \frac{d [\!\text{ROR\(\gamma\)t}_{\text{mRNA}}] }{d t} = &\quad \theta_{10} [\!\text{TGF\(\beta\)}_{\text{int}}] [\!\text{STAT3}_{\text{prot}^{*}}] \end{array} $$

(16) $$\begin{array}{*{20}l} & -\theta_{11} [\!\text{FOXP3}_{\text{prot}^{*}}] [\!\text{ROR\(\gamma\)t}_{\text{mRNA}}] \end{array} $$

(17) $$\begin{array}{*{20}l} & -\theta_{12} [\!\text{ROR\(\gamma\)t}_{\text{mRNA}}], \end{array} $$

where *θ*_10_, *θ*_11_, and *θ*_12_ are unknown rate constants (see Table 2).

Within our model, we allow FOXP3 activation to be regulated by the dynamic intracellular TGF *β* level and by basal mechanisms. In addition, we allow Th17 lineage specific signals to inhibit FOXP3 expression through STAT3 phosphoprotein. These mechanisms are described by the ODEs

(18) $$\begin{array}{*{20}l} \frac{d [\!\text{FOXP3}_{\text{mRNA}}] }{d t} &=\quad \theta_{13} \end{array} $$

(19) $$\begin{array}{*{20}l} &\quad +\theta_{14} [\!\text{TGF\(\beta\)}_{\text{int}}] \end{array} $$

(20) $$\begin{array}{*{20}l} &\quad -\theta_{15} [\!\text{STAT3}_{\text{prot}^{*}}] [\!\text{FOXP3}_{\text{mRNA}}] \end{array} $$

(21) $$\begin{array}{*{20}l} &\quad -\theta_{16} [\!\text{FOXP3}_{\text{mRNA}}] \end{array} $$

(22) $$\begin{array}{*{20}l} \frac{d [\!\text{FOXP3}_{\text{prot}^{*}}] }{d t} &=\quad \theta_{17} [\!\text{FOXP3}_{\text{mRNA}}] \end{array} $$

(23) $$\begin{array}{*{20}l} &\quad - \theta_{18} [\!\text{FOXP3}_{\text{prot}^{*}}], \end{array} $$

where *θ*_13_−*θ*_18_ are unknown rate parameters (see Table 2). We assume that the initial level of FOXP3 protein is non-zero. Without losing any generality, we treat $[\!\text {FOXP3}_{\text {prot}^{*}}]$ as a unitless quantity and set its initial level to equal one. Initial values for all mRNA levels are taken from RNA-seq measurement (mean abundance of three replicates at time 0 h). Other initial values are as stated above.

All model parameters are defined to be strictly positive, and to introduce prior knowledge on the parameter ranges, we use standard normal prior distributions in logarithmic parameter space and assume that a priori dependencies between the parameters do not exist. The model was also implemented in COPASI [25] to generate Systems Biology Markup Language (SBML) encoded version of the model. The SBML encoded version can be found in Additional file 2.

### Linking mathematical model with RNA-seq data

In order to combine our mathematical model with time-course RNA-seq measurements, we need to construct a statistical model that is capable of integrating continuous-state model responses with discrete count data. The traditional way of doing this would be to carry out some kind of ad hoc normalization for the count data and then fit the model responses to the normalized data using for example least squares or maximum likelihood methods. However, this kind of approach can be detrimental for many reasons. First, we do not typically know the statistical properties of the normalized count data and assuming a normal distribution for normalized count data might in some cases corrupt the analysis [26, 27]. Second, even if we knew the exact statistical model for the normalized data, we might not be able parameterize it. To prevent these kinds of problems when combining our mathematical model with count data, we make use of the negative binomial (NB) distribution which is also used in state-of-the-art statistical data analysis methods for sequencing data [24, 28].

Let us consider an arbitrary (time-course) RNA-seq data set that is organized in a three dimensional matrix *D* so that each element *D*_*ijk*_, (*i*=1,…,*n*;*j*=1,…,*m*;*k*=1,…,*l*) represents the read count of gene *i* at the *j*th time point *t*_*j*_ in the *k*th replicate. We model these data as NB distributed and using the same notation as Robinson et al. [24], we can write

(24) $$ {D}_{ijk} \sim \text{NB}(L_{jk}p_{ij},\phi_{ij}),  $$

where *L*_*jk*_ is the library size, *ϕ*_*ij*_ is the gene specific, time-dependent dispersion parameter, and *p*_*ij*_ is the relative mRNA abundance of gene *i* at time *t*_*j*_. The parameterization which Robinson et al. [24, 29] use lets us also express the mean in the form *μ*_*ij*_=*L*_*jk*_*p*_*ij*_ and variance in the form *μ*_*ij*_(1+*ϕ*_*ij*_*μ*_*ij*_).

Let us now consider the output of a mathematical model, $\mathbf {x}(t,\boldsymbol {\theta })\in \mathbb {R}_{\geq 0}^{n}$ that describes the relative mRNA abundancies for *n* genes as a function of time *t*. Here, all model parameters are collected into a vector $\boldsymbol {\theta }\in \mathbb {R}^{d}$. By assuming independent measurements and denoting *x*_*ij*_(***θ***)=*x*_*i*_(*t*_*j*_,***θ***), the likelihood of reproducing the observed data using the model can now be defined as

(25) $$ p(D|\boldsymbol{\theta}) = \prod_{i=1}^{n} \prod_{j=1}^{m} \prod_{k=1}^{l} g(D_{ijk};L_{jk}x_{ij}(\boldsymbol{\theta}),\phi_{ij}),  $$

where *g*(·;*μ*,*ϕ*) is the probability mass function of NB distribution with the mean *μ* and dispersion parameter *ϕ*. The function *g* can be written in the form

(26) $$ g(y;\mu,\phi) = \frac{\Gamma(y + \phi^{-1})}{\Gamma(\phi^{-1})\Gamma(y + 1)} \left(\frac{1}{1 + \mu\phi} \right)^{\phi^{-1}} \left(\frac{\mu}{\phi^{-1} + \mu} \right)^{y}  $$

giving the variance *μ*+*ϕμ*^2^ [30, 31].

### Statistical framework

Bayesian methodology offers a powerful formalism to carry out parameter inference, model discrimination, and experimental design for mathematical models (see e.g. [32–36]). In this study, we also adapt the Bayesian approach. According to Bayes’ theorem, the parameter posterior distribution takes the form *p*(***θ***|*D, M*)∝*p*(*D*|***θ***,*M*)*p*(***θ***|*M*), where ***θ*** is a vector of parameters, *M* denotes the model of interest, *D* denotes the observed data, and *p*(***θ***|*M*) is a model specific prior distribution for the parameters (for details about Bayesian data analysis, see e.g. [37]). As we have already defined the likelihood function *p*(*D*|***θ***,*M*) to combine mathematical models with RNA-seq data, the statistical tools provided by Bayesian methodology are available for us in their full power.

### Model discrimination

In the above notation, we have written the densities conditional to a specific model *M*. The reason for this is that we are interested in considering alternative models and, more specifically, evaluating the evidence for alternative models given the observed data. The evidence (marginal likelihood) for a given model *M* is

(27) $$ p(D|M) = \int_{\boldsymbol{\theta}\in\Theta} p(D|\boldsymbol{\theta},M)p(\boldsymbol{\theta}|M) d\boldsymbol{\theta}.  $$

If we consider a set of alternative models *M*_*i*_,*i*=1,…,*N* and do not assume any a priori preference for any of the models, the marginal likelihoods *p*(*D*|*M*_*i*_) can be directly used to assess the evidence for different models. In other words, the higher the marginal likelihood, the more evidence the data provides for that particular model. This approach also inherently penalizes overly complex models and overfitting (for details, see e.g. [38]).

### Posterior predictions

The posterior predictive distribution of a continuous-time model response is defined by writing

(28) $$ p(\mathbf{y}^{*}(t)|D,M) = \int_{\boldsymbol{\theta}\in\Theta} p(\mathbf{y}^{*}(t)|\boldsymbol{\theta},M)p(\boldsymbol{\theta}|D,M)d\boldsymbol{\theta},  $$

where $\mathbf {y}^{*}(t)=(y^{*}_{1}(t),\dots,y^{*}_{n}(t))\in \mathbb {R}_{\geq 0}^{n}$ is the predicted model output at time *t*. However, this formulation cannot be applied directly because we have defined the likelihood function *p*(**y**^∗^(*t*)|***θ***,*M*) only for the observed points in the space of model output. Consequently, we need to define an interpolated version of the likelihood function that can be used to estimate the posterior predictive distributions in continuous-time. The interpolated version of the likelihood function for this purpose is defined by

(29) $$ \bar{p}(\mathbf{y}^{*}(t)|\boldsymbol{\theta},M) = \prod_{i=1}^{n} g(\bar{L}y_{i}^{*}(t);\bar{L}x_{i}(t,\theta),\bar{\phi}_{i}(t)),  $$

where $\bar {L}$ is the average library size estimated from the data and $\bar {\phi }_{i}(t)$ is a continuous time dispersion parameter interpolated from the original estimated dispersion parameters.

### Thermodynamic integration

The estimation of marginal likelihood is challenging in general and there exists several numerical techniques for this purpose (for a review, see [39, 40]). Thermodynamic integration is one of the most powerful approaches proposed and it has also been successfully applied in the context of mathematical models (see e.g. [33, 34, 41, 42]). To estimate marginal likelihood by means of thermodynamic integration, we need to define the so-called power posterior distribution

(30) $$ p_{\beta}(\boldsymbol{\theta}|D,M) \propto p(D|\boldsymbol{\theta},M)^{\beta}p(\boldsymbol{\theta}|M),  $$

where *β*∈ [ 0,1]. Clearly, the power posterior distribution can be used to define a discrete set of bridging distributions between the prior and posterior distribution. Further, it can be shown (see e.g. [39]) that

(31) $$ \ln(p(D|M)) = {\int_{0}^{1}} \left[\! \int \ln(p(D|\boldsymbol{\theta},M)) p_{\beta}(\boldsymbol{\theta}|D,M) d\boldsymbol{\theta} \right] d\beta  $$

and this allows us to define the estimator

(32) $$ \widehat{\ln(p(D|M))} = \sum_{i = 2}^{N_{\beta}} (\beta_{i} - \beta_{i-1}) \left(\frac{I_{\beta_{i}} + I_{\beta_{i-1}} }{2}\right),  $$

where

(33) $$ I_{\beta_{i}} = \frac{1}{N_{s}}\sum_{j = 1}^{N_{s}} \ln(p(D|\boldsymbol{\theta}_{\beta_{j}},M)), \quad \boldsymbol{\theta}_{\beta_{j}} \sim p_{\beta_{i}}(\boldsymbol{\theta}|D,M)  $$

for some fixed $0 = \beta _{1} < \beta _{2} < \dots < \beta _{N_{\beta }} = 1$, where *N*_*β*_ and *N*_*s*_ are the number of bridging distributions and the number of samples from each distribution, respectively. To compute $I_{\beta _{i}}$, *i*=1,…,*N*_*β*_, we need samples from the bridging distributions $p_{\beta _{i}}(\boldsymbol {\theta }|D,M)$. A very convenient way of obtaining these samples is to use population-based Markov Chain Monte Carlo sampling.

### Population-based markov chain monte carlo

The performance of optimization and sampling techniques is often hampered by complex dependencies between parameters as well as multi-modality of the target distribution. Population-based Markov Chain Monte Carlo (MCMC) sampling algorithms of various types have been developed to enable sampling also from these kinds of complex distributions [43]. In this study, we construct a population-based MCMC sampling algorithm that serves two purposes. First, we need a sampler that is capable of sampling from complex multimodal distributions and, second, the sampler should provide us with samples that can be used to estimate the marginal likelihood by means of thermodynamic integration.

We construct our population-based MCMC algorithm according to the guidelines given by Friel and Pettit [39] and Calderhead and Girolami [41], and define a product form of the target density

(34) $$ p^{*}(\boldsymbol{\theta}_{\beta_{1}},\boldsymbol{\theta}_{\beta_{2}},\dots,\boldsymbol{\theta}_{\beta_{N_{\beta}}}|D,M) = \prod_{i = 1}^{N_{\beta}} p_{\beta_{i}}(\boldsymbol{\theta}_{\beta_{i}}|D,M),  $$

where $0 = \beta _{1} < \beta _{2} < \dots < \beta _{N_{\beta }} = 1$ and $p_{\beta _{i}}$ are as defined above. The product form of target density has all bridging distributions as its marginal distributions and we draw samples from each of these distributions in parallel. In other words, our sampler runs *N*_*β*_ parallel MCMC chains, one chain sampling from each $p_{\beta _{i}}, i = 1,\dots,N_{\beta }$ and in addition to local exploration of the target distribution we allow the parallel chains to exchange information through global moves between the chains. A detailed description of our sampling algorithm can be found in Additional file 1 and an example implementation can be found in Additional file 3.

### Computational implementation

We implement the mathematical models and sampling algorithm in Matlab (The MathWorks Inc., Natick, MA, USA) and use ode15s solver to numerically solve ordinary differential equation systems. We use 30 temperatures (i.e. *N*_*β*_=30) to carry out population-based MCMC sampling and to discretize the thermodynamic integral. To construct an efficient sampling algorithm, the bridging distributions must allow free movement of MCMC chains close to the prior distribution and, on the other hand, the bridging distributions should be appropriately related to enable efficient information exchange between temperatures. Consequently, the selection of values for $\beta _{2} < \dots < \beta _{N_{\beta }-1}$ plays an important role in the efficiency of the algorithm and the accuracy of estimates computed based on the samples. In this study, we set *β*_*j*_=((*j*−1)/(*N*_*β*_−1))^5^*j*=1,…,*N*_*β*_ and initially run the sampler using a symmetric normal proposal distribution. After approximate convergence is obtained, we tune the proposal distributions based on the observed covariances of samples and the final sample is then generated using the tuned, fixed proposal distributions. For each model we run five independent samplers, collect every 1000th sample, and the convergence of Markov chains is monitored using the potential scale reduction factors [37] (see Additional file 1: Figures S1 and S2) as well as visual inspection of log-likelihood and sample traces. The final MCMC sample size for each model was 10,000 samples before the incorporation of FOXP3 protein data and 5000 samples after the incorporation of these data.

## Results

### Dynamic description is consistent with data

In the previous section, we proposed a dynamic description for the core regulatory network steering Th17 cell differentiation and converted this description into a mathematical model. The dynamic description includes four hypothetical mechanisms and, by considering different combinations of these mechanisms, we chose to study 12 alternative scenarios for molecular interactions (see Fig. 1 and Table 1). To quantitatively assess if these alternative scenarios are in agreement with experimental data, we combined the corresponding mathematical models with time-course RNA-seq measurements using our framework for sequencing data and carried out statistical inference using the methodology described above. As a result of the statistical analysis, we conclude that our numerical methods work well and that all alternative models are identifiable (the parameter posterior distributions get notably updated when compared with our uninformative standard normal prior distributions in log-scale, see Additional file 1: Figures S3 and S4). Further, by observing the posterior predictive distributions over the mRNA dynamics it is easy to conclude that all alternative models are capable of describing the dynamics observed in the experimental data (Additional file 1: Figure S5). Consequently, it is not possible to evaluate the alternative models solely by visual inspection and we need to utilize statistical testing to quantitatively assess the evidence for different models.

### Statistical testing shows strong evidence for inhibitory mechanisms

**Fig. 2:**
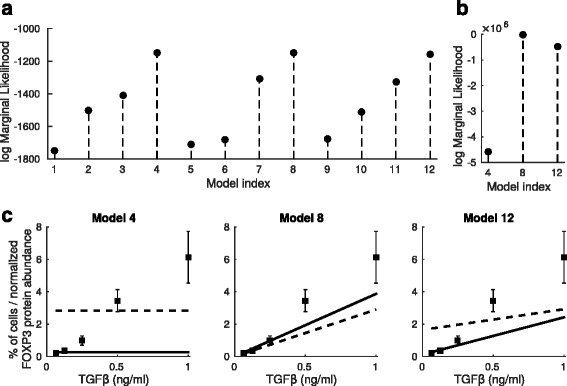
Estimated evidence for alternative models and comparison of model predictions with experimental data. **a** Estimated marginal likelihoods for 12 alternative models. **b** Estimated marginal likelihoods for highly ranked models (Models 4, 8, and 12) after incorporation of additional data on FOXP3 protein levels. **c** Experimental data on FOXP3 protein levels (the percentage of cells expressing FOXP3 protein at time 72 h) plotted with the corresponding predicted average FOXP3 levels as a function of added TGF *β* (predictions are generated using Models 4, 8, and 12, and the dimensionless latent FOXP3 level is multiplied by 10^4^ for illustrative purposes). The experimental data (black squares) shown as mean ± standard deviation are representative of 3 independent experiments. The dashed and solid lines represent the predictions generate before and after incorporation of FOXP3 protein level data, respectively

We estimate the evidence (marginal likelihood) for each model to quantitatively evaluate the alternative models. Results show that Models 4, 8, and 12 have clearly higher evidence than the other models (Fig. 2a). The common feature of the highly ranked models is that they all include both hypothetical inhibitory mechanisms, ROR *γ*t inhibition by FOXP3 and FOXP3 inhibition by STAT3. Further, Model 4 which has the highest evidence does not allow the dependency between TFG *β* and FOXP3. In biological terms, this would suggest that in Th17 polarizing condition the level of TFG *β* is so low that it does not affect the FOXP3 expression at all. To experimentally validate this prediction, we quantified the FOXP3 protein abundance at time 72 h in Th17 polarization conditions using five different concentrations of added TGF *β* that were diluted with respect to the original dose. Surprisingly, these data show that FOXP3 protein expression depends on the TGF *β* dose in Th17 polarizing conditions (Fig. 2c). To test if any of the highly ranked models (Models 4, 8, and 12) is capable of reproducing this behavior, we generated the corresponding posterior predictive distributions for FOXP3 levels at 72 h and plotted the estimated mean FOXP3 protein levels against the data (dashed lines in Fig. 2c). Because Model 4 allows only constant basal activation of FOXP3, it is naturally not capable of reproducing the dependency that is seen in experimental data. On the other hand, Models 8 and 12 predict similar trend as seen in the data (Fig. 2c). Our conclusion here is that the time-course RNA-seq data shows significant evidence for the hypothetical inhibitory mechanisms but to be able to make any conclusions about the FOXP3 activation mechanisms and its dependency on the amount of added TGF *β*, we need to incorporate also the additional data on FOXP3 levels into our statistical framework and repeat the statistical testing.

### Incorporation of FOXP3 protein data approves the inhibitory mechanisms and FOXP3 dependence on TGF *β*

**Fig. 3:**
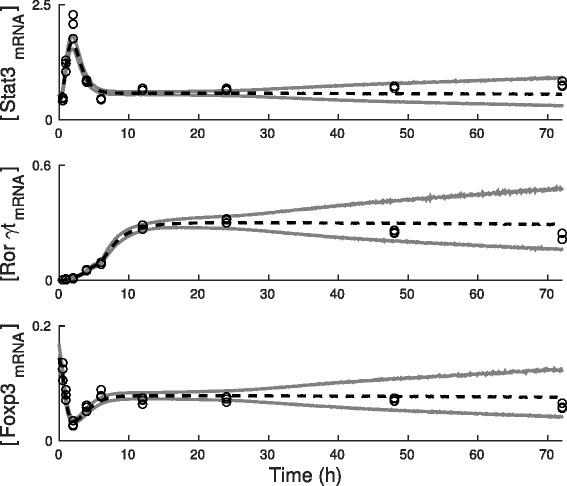
Posterior predictive distributions generated using the Model 8 after the incorporation of FOXP3 protein data. Time-dependent marginal posterior predictive distributions for [STAT3 _mRNA_], [ROR *γ* *t*_mRNA_], and [FOXP3 _mRNA_] are illustrated using the estimated 5 *%* and 95 *%* percentiles (grey lines) and the median (dashed line). The data are plotted using circles. The data are normalized by dividing each value by the corresponding library size and the scaling constant that is used in the model

To incorporate the dilution experiment data on FOXP3 levels into our statistical framework, we extend the likelihood function by multiplying it by distributions for observed protein levels in different dilutions. These distributions are taken to be normal distributions that have the scaled predicted protein level as their mean and the variance is estimated from observed protein levels (i.e. the variance corresponds to the standard deviations shown in Fig. 2c). Having extended the likelihood function, we can repeat the estimation of evidence for the models 4, 8, and 12 using the full data that includes also the protein measurements. The updated estimated marginal likelihoods are shown in Fig. 2b and, as supposed, Model 4 is not highly ranked when the dependency between FOXP3 and TGF *β* is taken into account in the statistical testing. Instead, Model 8 has now the highest evidence and Fig. 2c (solid lines) shows that also the predictions generated by the Model 8 are in a better agreement with the data than the predictions generated by Models 4 and 12. We conclude here that based on our statistical analysis Model 8 has the highest evidence meaning that our data supports the existence of the both inhibitory interactions in the model. In addition, our results show, in agreement with validation data, that FOXP3 protein level depends on TGF *β* level during Th17 lineage specification. Figure 3 shows the estimated posterior predictive distributions for the mRNA levels given the full data containing the both RNA-seq and FOXP3 protein measurements. The posterior predictive distributions are in a good agreement with the observed mRNA abundances.

## Discussion and conclusions

While the cytokines that induce naive CD 4^+^ T cells to differentiate into specific subsets are well known, the mechanistic detailed interactions between the key transcription factors are not known. As a comprehensive experimental determination of the detailed dynamics is out of reach, mathematical modeling is an indispensable aid to learn more about the differentiation processes. In this study, we observe Th17 specific transcription factor dynamics through time-course mRNA data (RNA-seq measurements) and use objective computational tools to learn the mechanistic interactions of the key transcription factors. By integrating the mathematical models with our comprehensive set of time-course RNA-seq data as well as qualitative information about the differentiation dynamics, we construct an experimentally-based dynamic model that is capable of producing meaningful predictions that we also validate experimentally.

We model the Th17 differentiation process on the same level as the process is typically experimentally observed. Our modeling approach is unique in three ways. First, we use time-course RNA-seq data as a basis of our work and integrate the data with dynamic modeling using a framework that makes use of statistical characterization of sequencing data. Second, we apply powerful statistical methodology that allows us to evaluate the alternative model topologies and, even more importantly, allows us to estimate the posterior predictive distributions for any process included into the model. Because we have a statistical model for the experimental data, the predictive distributions conveniently reflect the uncertainty in the observations as well as in the structure of our models. Third, we can generate probabilistic predictions in a continuous range of cytokine conditions on contrary to just knocking down a gene or removing a cytokine input from the model. In other words, we are able to make predictions in a continuous range of cytokine conditions (e.g. different dilution levels for TGF *β*, see Fig. 2).

Previously published models for Th17 differentiation have been a part of larger studies which have aimed at modeling CD 4^+^ T cell differentiation into various subsets [17, 18, 22] or have been especially designed to describe the reciprocal differentiation into Th17 and iTreg cells [20, 21]. Although these studies provide us with valuable information about the Th17 cell differentiation process, the models do not allow efficient use of time-course data and, more importantly, the model structure cannot be quantitatively judged by experimental observations.

The applicability of our modeling approach is not restricted to the modeling of Th17 cell differentiation. The statistical framework that we develop to combine mathematical models with sequencing data is fully general and can be used to analyze any RNA-seq data given an adequate continuous-state dynamic model. Our framework incorporates all available information on the statistical properties of sequencing data into the likelihood function which has the form of the negative binomial (NB) distribution. The NB distribution is also used in state-of-the-art data analysis methods [24, 28] and, thus, all important features of data, such as time-dependent gene-wise over-dispersion, can be automatically taken into account when constructing the likelihood function. Further, the NB likelihood function conveniently links the discrete read count data with continuous-state dynamic models. The NB distribution is unarguably the most well suited distribution for read count data [24, 28] and, for this reason, our framework is inherently better suited for linking dynamic models with sequencing data than standard methods such as maximum likelihood fit via normal distribution. In some parameter regimes, the NB distribution can be approximated using the normal distribution and, in these cases, maximum likelihood fit might also well approximate the best fit obtained from our rigorous model. However, to construct this kind of normal approximations, one still needs to extract information from the sequencing data to incorporate, for example, time-dependent features and, importantly, one needs to check that the normal approximation is valid in all ranges of data at all time points considered. In general, normal approximations for count data are noted to perform poorly [26, 27] and by means of our framework, these kinds of cumbersome approximations can be simply avoided.

In summary, we introduce an experimentally-based modeling approach for Th17 cell differentiation and use it to generate predictions that are experimentally validated. The modeling is carried out within a novel statistical framework and, by using statistical methodology, we carry out experimentally based construction of a mathematical model. Our study approves the important role of quantitative dynamic modeling in identifying and studying regulatory mechanisms. Advanced application of mathematical modeling takes the analysis of time-course data well beyond the standard statistical analyses and offers powerful means to test the existing knowledge against data as well as to hypothesize alternative mechanisms that can generate the observed data. There are two central requirements for successful application of mathematical modeling to these kinds of analysis tasks. First, the model construction process needs to be data and knowledge driven and, second, the resulting models must be suitable for quantitative evaluation. Our experimentally-based modeling framework for Th17 cell differentiation is designed to serve these needs in the best possible way and can be regarded powerful in the sense that it enables us to fully account for the uncertainty in the data as well in the model structure.

## Ethics Statement

All animal experiments were done according to institutional guidelines and Home Office regulations. The experimental protocols for work with animals have been approved by the MRC National Institute for Medical Research (now The Francis Crick Institute) ethical review board and the respective national regulatory agencies (Home office Licence: PPL 80/2506).

## Availability of supporting data

The data sets supporting the results of this article are included within the article and its additional files.

## Additional files

Additional file 1
**Supplementary information**. The supplementary information consists of three sections 1. Population-based Markov chain Monte Carlo, 2. RNA-seq data, and 3. Supplementary Figures. (PDF 837 kb)

Additional file 2
**Mathematical model encoded in SBML**. (XML 28 kb)

Additional file 3
**Implementation of population-based Markov chain Monte Carlo in Matlab**. (ZIP 46 kb)
