# Supplementary material for: Analyzing Th17 cell differentiation dynamics using a novel integrative modeling framework for time-course RNA sequencing data
Source: BMC Syst Biol. 2015 Nov 17;9:81. doi: 10.1186/s12918-015-0223-6 (PMC4650136; doi:10.1186/s12918-015-0223-6)
Supplement: Additional file 1 — Supplementary information. The supplementary information consists of three sections 1. Population-based Markov chain Monte Carlo, 2. RNA-seq data, and 3. Supplementary Figures. (PDF 837 kb) [file 12918_2015_223_MOESM1_ESM.pdf]

Supplementary information

Analyzing Th17 cell differentiation dynamics using a novel integrative modeling framework for time-course RNA sequencing data

Contents

1 Population-based Markov Chain Monte Carlo **1**

2 RNA-seq data **2**

3 Supplementary Figures **4**

1 Population-based Markov Chain Monte Carlo

Let us consider N_β bridging distributions

$$p_{\beta_i}(\theta_{\beta_i}|y) \propto p(y|\theta_{\beta_i})^{\beta_i} p(\theta_{\beta_i}), \tag{1}$$

where $0 = \beta_1 < \beta_2 < \cdots < \beta_{N_\beta} = 1$ and $\theta_{\beta_i} \in \mathbb{R}^d$. Population-based Markov Chain Monte Carlo sampler can now be constructed by defining a new target distribution as a product of these distributions i.e.

$$p^*(\theta_{\beta_1}, \theta_{\beta_2}, \ldots, \theta_{\beta_{N_\beta}}|y) = \prod_{i=1}^{N_\beta} p_{\beta_i}(\theta_{\beta_i}|y). \tag{2}$$

and running N_β parallel samplers in the corresponding marginal distributions $p_{\beta_i}(\theta_{\beta_i}|y)$. We implement our population-based MCMC sampling procedure by defining two kinds of moves: (i) local moves within distinct distributions and (ii) global moves that allow swapping between neighboring distributions (confer e.g. [1, 2]). In the following, we refer to the bridging distributions using the fixed values of β_i that are called temperatures.

Local move at temperature β_j: Given the current state of the chain $\theta = (\theta_{\beta_1}, \theta_{\beta_2}, \ldots, \theta_{\beta_j}, \ldots, \theta_{\beta_{N_\beta}})$, we propose a new state $\theta^* = (\theta_{\beta_1}, \theta_{\beta_2}, \ldots, \theta^*_{\beta_j}, \ldots, \theta_{\beta_{N_\beta}})$ where $\theta^*_{\beta_j}$ is drawn from a normal proposal distribution which is centered at the current state and has a temperature specific, constant covariance matrix $\Sigma_{\beta_i} \in \mathbb{R}^{d \times d}$. In other words, $\theta^*_{\beta_j} \sim \mathrm{N}(\theta_{\beta_j}, \Sigma_{\beta_i})$. The proposed state can then be accepted with probability $\min(1, r)$, where $r = p(y|\theta^*_{\beta_j})^{\beta_j} p(\theta^*_{\beta_j}) / p(y|\theta_{\beta_j})^{\beta_j} p(\theta_{\beta_j})$ according to the standard Metropolis-Hastings acceptance ratio with a symmetric proposal distribution [2].

Global moves between neighboring temperatures β_j and β_{j+1}: Given the current state of the chain $\theta = (\theta_{\beta_1}, \theta_{\beta_2}, \ldots, \theta_{\beta_j}, \theta_{\beta_{j+1}}, \ldots, \theta_{\beta_{N_\beta}})$, we propose a new state $\theta^* = (\theta_{\beta_1}, \theta_{\beta_2}, \ldots, \theta^*_{\beta_j}, \theta^*_{\beta_{j+1}}, \ldots, \theta_{\beta_{N_\beta}})$, where $\theta^*_{\beta_j} = \theta_{\beta_{j+1}}$ and $\theta^*_{\beta_{j+1}} = \theta_{\beta_j}$. The proposed state can then be accepted with probability $\min(1, r)$, where $r = p(y|\theta_{\beta_{j+1}})^{\beta_j} p(y|\theta_{\beta_j})^{\beta_{j+1}} / p(y|\theta_{\beta_j})^{\beta_j} p(y|\theta_{\beta_{j+1}})^{\beta_{j+1}}$ according to the standard Metropolis-Hastings acceptance ratio with a symmetric proposal distribution [2].

In our implementation, one iteration of the sampler consists two steps. In the first step, a temperature index j is sampled from the uniform distribution $U(1, N_\beta)$ and a local move at the temperature β_j is

proposed. In the second step, global moves between all neighboring temperatures are proposed starting from the prior distribution and, once the posterior distribution is reached, the scan is repeated backwards towards the prior distribution to restore the reversibility of the chain. The combination of these local and global proposal moves results in a time homogeneous transition kernel that has the target distribution p^* as its stationary distribution. A Matlab implementation of the sampler is available via the web page http://research.ics.aalto.fi/csb/software.

2 RNA-seq data

Time (h)	STAT3	RORγt	FOXP3	Lib. size
Replicate 1				
0	6807	9	2238	16410829
0.5	8413	7	1869	17657582
1	20556	6	1139	15875968
2	43406	146	485	18991346
4	14371	902	836	16496678
6	7566	1545	1233	16118519
12	12123	5066	1133	17493780
24	10904	5313	1268	16432535
48	12619	4541	1336	18280419
72	17865	4989	1526	23446761
Replicate 2				
0	4893	55	1607	10804012
0.5	5006	41	1493	11926410
1	15971	40	1046	13055612
2	31584	223	420	15109892
4	13910	998	1016	17119113
6	7049	1531	1253	15979147
12	9581	3972	1063	14852672
24	10807	5133	1155	16999116
48	12765	4407	1201	17429518
72	17548	5074	1208	20762331
Replicate 3				
0	8686	38	2580	17192850
0.5	7545	73	2257	16581768
1	20473	108	1732	19445890
2	22646	149	440	12841317
4	12129	703	880	14228551
6	7267	1316	1427	15986800
12	11774	4688	1292	17346748
24	13016	5978	1405	19052670
48	14390	5632	1588	21273105
72	15932	4568	1222	21587524

Time (h)	Dispersion, ϕ
0.5	0.0017415661
1	0.0027156614
2	0.0020994483
4	0.0020447991
6	0.0011874179
12	0.0007739092
24	0.0030286538
48	0.0437877137
72	0.1037737656

3 Supplementary Figures

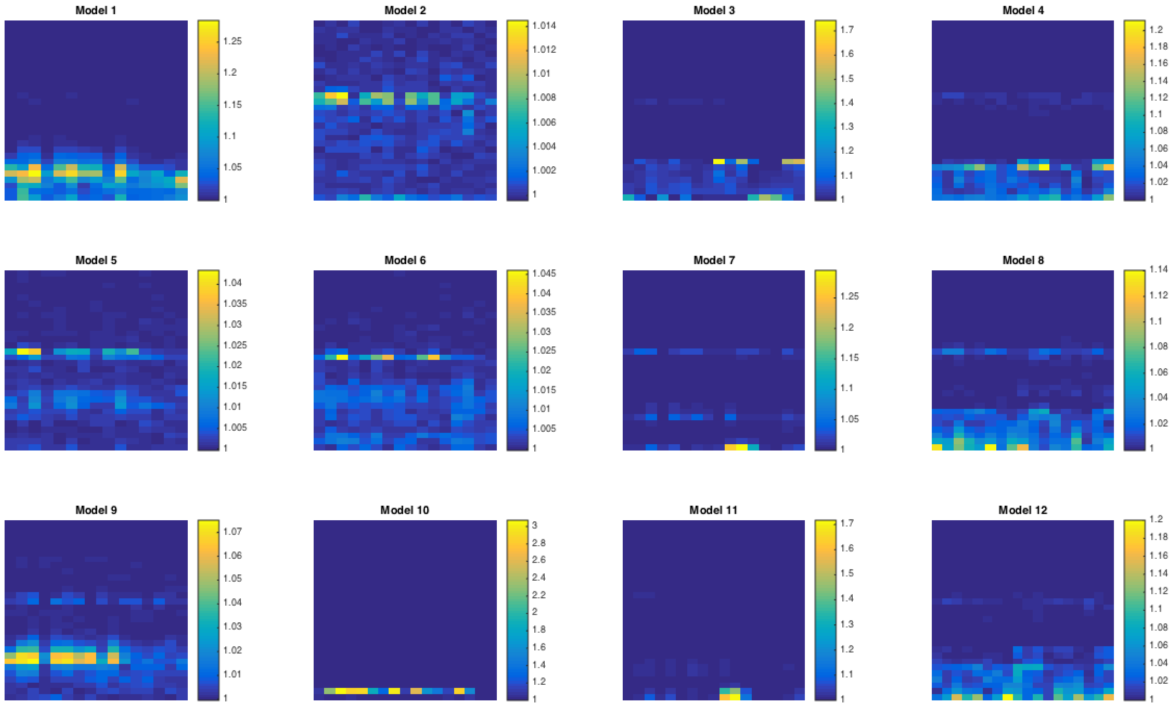

Figure S1 Potential scale reduction factors (PSRFs) for the sampling results with the RNA-seq data. Each subplot shows PSRFs for all parameters of the particular model (columns) in all 30 temperatures (rows). Based on PSRFs and visual inspection of the log-likelihood and sample traces the chains have converged.

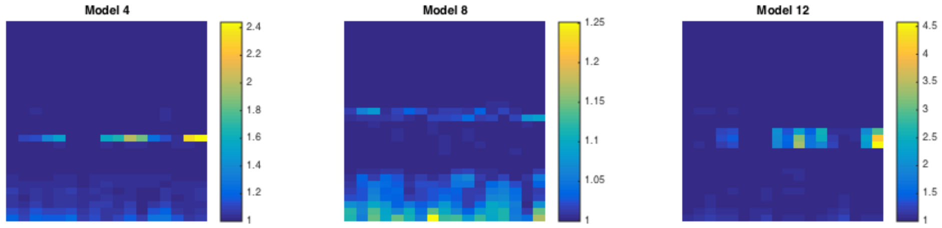

Figure S2 PSRFs for the sampling results after incorporation of FOXP3 protein data.

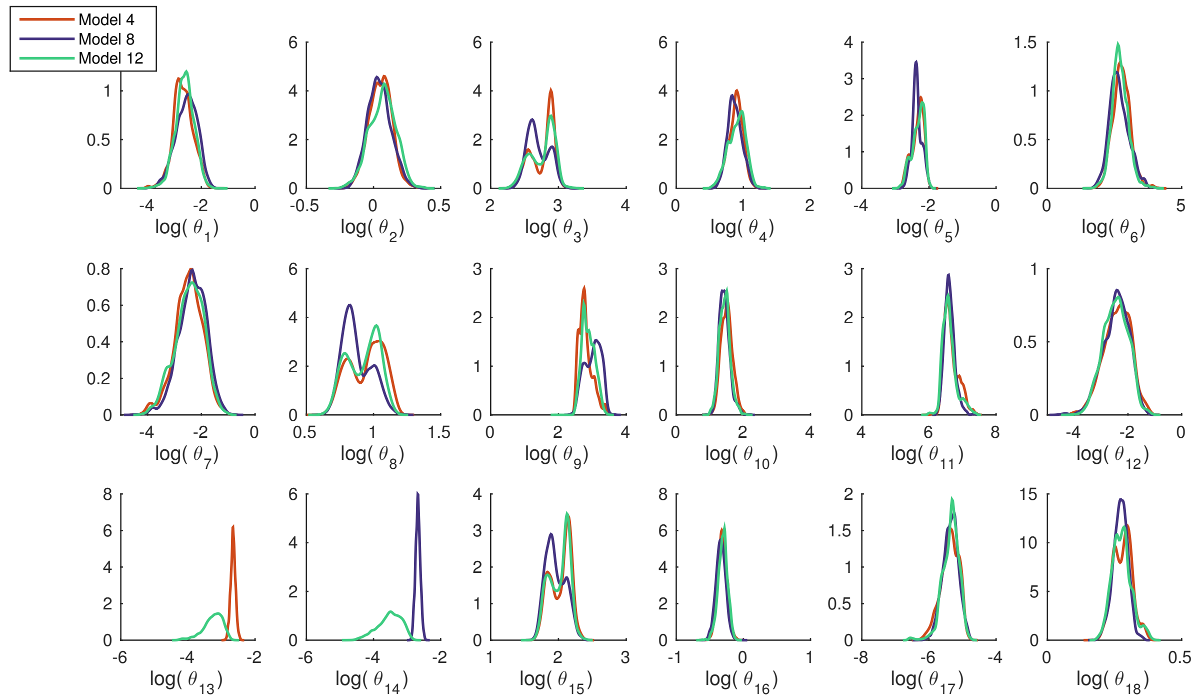

Figure S3 Estimated marginal parameter posterior distributions for Models 4, 8, and 12 before the incorporation of FOXP3 protein data. The distributions are obtained from Markov chain Monte Carlo samples by using the kernel smoothing function estimate (ksdensity) implemented in Matlab®.

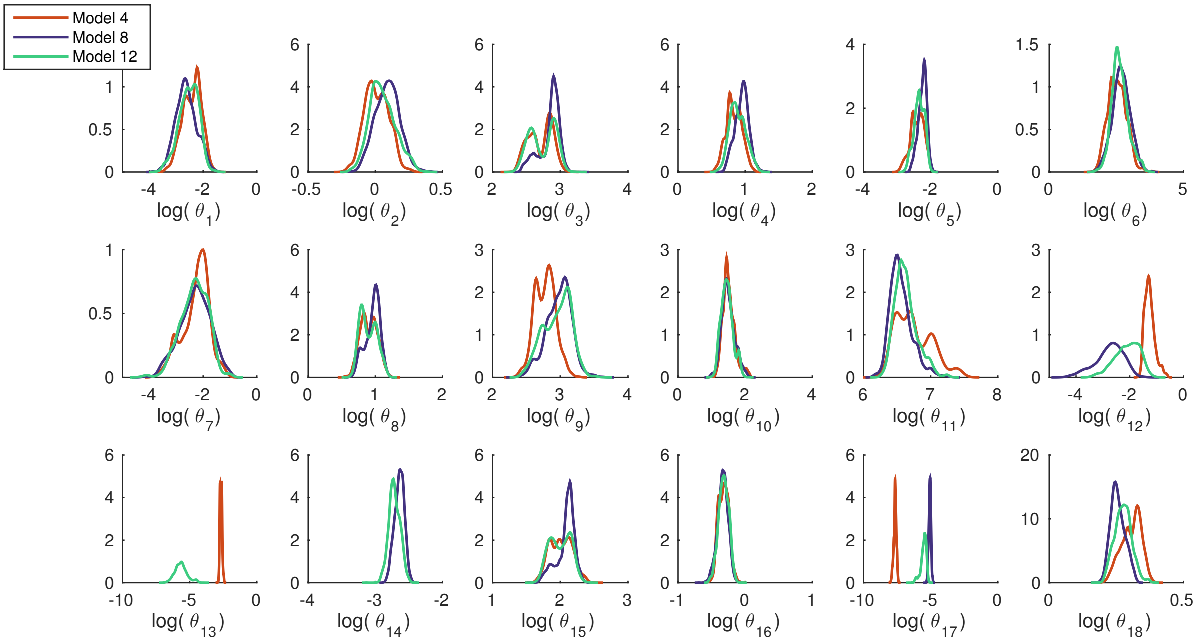

Figure S4 Estimated marginal parameter posterior distributions for Models 4, 8, and 12 after the incorporation of FOXP3 protein data. The distributions are obtained from Markov chain Monte Carlo samples by using the kernel smoothing function estimate (ksdensity) implemented in Matlab®.

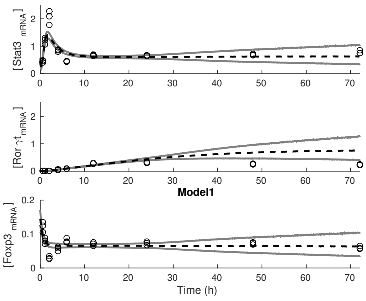

(a) Model 1

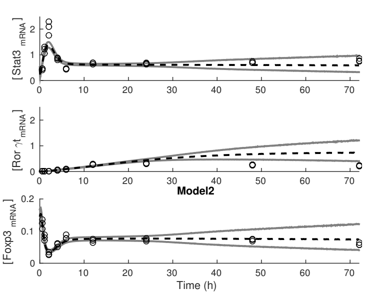

(b) Model 2

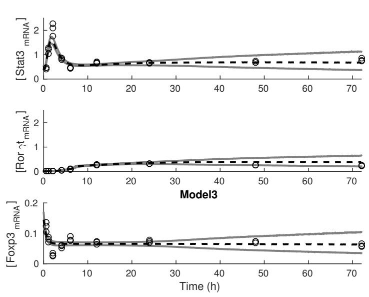

(c) Model 3

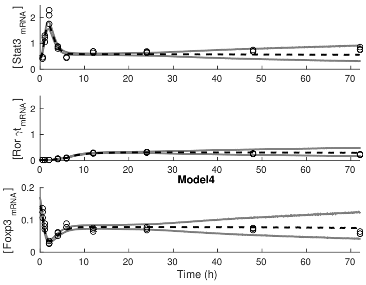

(d) Model 4

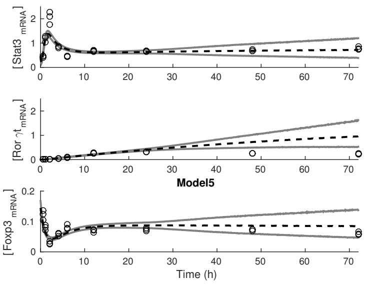

(e) Model 5

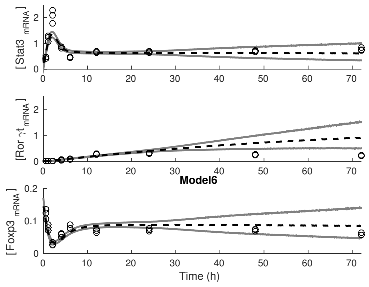

(f) Model 6

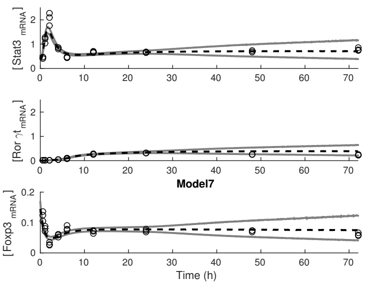

(g) Model 7

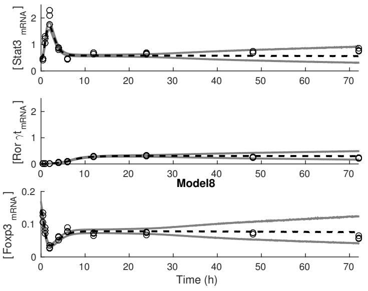

(h) Model 8

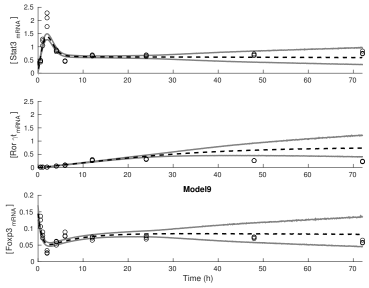

(i) Model 9

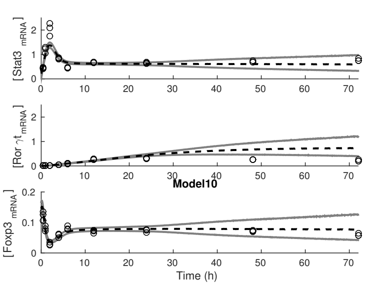

(j) Model 10

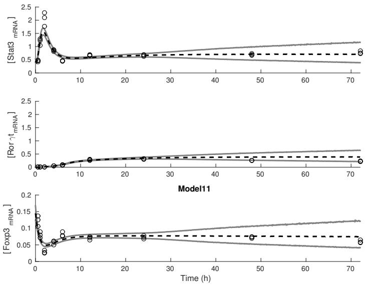

(k) Model 11

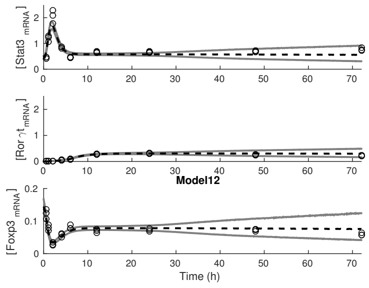

(l) Model 12

Figure S5 Marginal posterior predictive distributions generated using the Models 1 – 12 before the incorporation of FOXP3 protein data. Distributions are illustrated using the estimated 5% and 95% percentiles (grey lines) and the median (dashed line). The data are plotted using circles. The data are normalized by dividing each value by the corresponding library size and the scaling constant that is used in the model.

References

[1] Friel, N. and Pettitt, A. N. (2008) Marginal likelihood estimation via power posteriors. *J R Stat Soc Ser B Stat Methodol,* **70**(3), 589–607.

[2] Calderhead, B. and Girolami, M. (2009) Estimating Bayes factors via thermodynamic integration and population MCMC. *Comput Stat Data An,* **53**(12), 4028–4045.
